# Individualized Therapy Optimization for Type 2 Diabetes: Retrospective Study of Model Development, Internal Validation, and Physician Evaluation

**DOI:** 10.2196/92877

**Published:** 2026-10-09

**Authors:** Arina Lozhkina, Camillo Dario Piazza, Zeina Gabr, Maurice Rupp, David Herzig, Lia Bally, Andre Jaun

**Affiliations:** 1 Metadvice, Route Cantonale 109, St-Sulpice, Vaud, 1025, Switzerland; 2 Department of Diabetes, Endocrinology, Nutritional Medicine and Metabolism, University of Bern, Bern, Switzerland

**Keywords:** type 2 diabetes, artificial intelligence, AI, precision medicine, glucose-lowering pharmacotherapy, continuous glucose monitoring

## Abstract

**Background:**

Type 2 diabetes is a widespread chronic condition in which blood glucose and body weight management constitute essential therapeutic targets. Emerging technologies have the potential to aid complex therapeutic pharmacotherapy choices that are optimally tailored to individual needs.

**Objective:**

In this study, we developed and evaluated an AI model combining guidelines with clinical features and continuous glucose monitoring (CGM) to optimize therapeutic decision-making.

**Methods:**

Therapeutic guidelines were first encoded using a rule-based model and trained on a feed-forward neural network to predict the probability of therapeutic success for individual treatment recommendations. This approach relied on real-world evidence from a specialist diabetes outpatient clinic, using historical clinical data generated between 2009 and 2023. We used data from 533 patients with a diagnosis of type 2 diabetes and complete baseline data for weight and hemoglobin A_1c_ within relevant therapy windows, resulting in a total of 853 treatment regimens. Transfer learning was used to optimize for glucose-lowering therapies that led to successful treatment outcomes, defined as an absolute 0.3% reduction in hemoglobin A_1c_ (when it is over 6.5%) without weight gain in patients with a BMI over 28 kg/m^2^. Recommendations that deviated from the guidelines were described using Shapley values and tested in digital twins for statistical significance. Four CGM-derived glucose-insulin response dynamic factors served as additional biomarkers.

**Results:**

Dual glycemic and weight targets were achieved in actual clinical practice in 51.2% (131/256) of cases, increasing to 54% (20/37) when clinical guidelines were followed. After selecting outcomes in the test set that followed individualized recommendations, this increased further to 58% (21/36) when using only phenotypic markers and to 65% (22/34) when adding CGM-derived dynamic factors.

**Conclusions:**

Tested on the limited number of patients available, our findings show that our AI model was associated with improved retrospective outcomes compared to the guidelines in complex type 2 diabetes cases by integrating multiple data sources, drawing on experiential clinical insights, and selecting treatments most likely to meet each patient’s clinical targets for glucose and weight control. Future research is needed with a larger dataset.

## Introduction

Type 2 diabetes is a multifactorial disease with complex endocrine interactions and comorbidities. When lifestyle modifications are not sufficient, treatment involves glucose-lowering medication using different mechanisms of action, ranging from monotherapy to combination regimens. Given the high prevalence of overweight and obesity in patients with type 2 diabetes [1], glucose-lowering therapies should be selected to optimize both glycemic (hemoglobin A_1c_ [HbA_1c_]) and body weight control in most cases. Commonly used noninsulin pharmacotherapies to lower blood sugar include metformin as baseline treatment and, as an escalation, glucagon-like peptide-1 receptor agonists (GLP-1 RAs) and/or sodium-glucose cotransporter 2 inhibitors (SGLT2is), which have an additional beneficial effect on cardiorenal outcomes. If noninsulin pharmacotherapy is not sufficient to lower HbA_1c_, clinicians add insulin to the treatment, with the disadvantage of relevant side effects such as hypoglycemia or weight gain.

While clinical guidelines exist [2], multiple regimens are considered appropriate for outcome optimization, creating challenges in selecting the optimal individualized approach. Recent studies emphasize the need for precision medicine approaches that individualize treatment based on patient phenotypes, preferences, and therapeutic goals [3].

Several methods have been proposed to guide decision-making in type 2 diabetes. Therapy evaluation is typically designed as an emulation of a target trial aimed at population analysis rather than individual recommendations [4], for example, based on decision trees [5], autoregression [6], and gradient boosting methods [7]. In individualized treatment approaches, the clinical context is often limited, and optimization is guided solely by the reduction in HbA_1c_ [8,9].

More recent studies work with a limited set of treatment choices, for example, a machine learning model to select between GLP-1 RAs and SGLT2is [10]. Other studies have not compared different treatment options but have predicted whether a patient will respond to a treatment that has already been selected, such as GLP-1 RAs [11] or metformin [12]. Others have focused on reproducing prescribing rather than evaluating the effect of different treatments, for example, using a transformer model [13]. Another study evaluated ChatGPT by comparing its recommended antidiabetic treatments with clinicians’ prescriptions [14]. These approaches aimed to reproduce or approximate clinical treatment choices rather than estimate the individual treatment effect of several alternative therapies.

One prototype for decision support in type 2 diabetes [15] that incorporated dual optimization for both weight and glycemic control predicted changes in HbA_1c_ and weight for selected therapies; however, as the authors noted, clinical trial populations in the study did not fully reflect real-world treatment patterns and the diversity of patient populations.

Continuous glucose monitoring (CGM) sensors measure glucose values in the interstitial fluids. They enable patients and health care providers to obtain glucose values every 1 to 5 minutes. Compared to traditional values such as capillary-measured blood glucose or HbA_1c_, CGM data provide a granular assessment of glycemic patterns. By informing individual metabolic states and, consequently, the effect of treatment strategies, CGM has the potential to contribute to monitoring therapeutic responses in patients with type 2 diabetes [16,17]. Current research in the field is mostly focused on the stratification of patients to forecast the risk of complications and generally overlooks the potential to forecast therapeutic effects [18,19].

This work aimed to (1) develop and validate a neural network model using CGM as an input to optimize both weight and HbA_1c_ for patients in clinical settings and (2) evaluate the model’s recommendations through clinical specialists.

## Methods

### Model Development

Given the complexity of the decisions to be made and the relatively limited amount of data to learn, we first developed a rule-based neural network that reproduced commonly accepted guidelines with a high degree of accuracy. Historical records with phenotypic data that were available at the time of therapeutic decisions were then used with a transfer learning methodology in conjunction with real-world evidence outcomes to fine-tune the guideline knowledge and enrich training data with glucodynamics markers that were not available to clinicians. Relying on a separate test set, recommendations that deviated from guidelines were explained using Shapley values, defining similar digital twin cohorts so as to ensure that deviations from the guidelines were statistically significant in the retrospective analysis.

Clinical guidelines for the management of type 2 diabetes (European Association for the Study of Diabetes and American Diabetes Association [15]) were first enriched with expert knowledge from clinical practice, thereby reducing the risk of confounding when analyzing nonrandomized real-world data. These guidelines were used to create a rule-based model in the form of a decision tree relying on 61 input factors (age in years, weight, HbA_1c_ level, BMI, renal function [estimated glomerular filtration rate; eGFR], intolerance to specific drugs, current therapy, and glucose monitoring indicator) to choose 1 of 38 therapeutic pharmacological combinations (such as “metformin+GLP-1 RA”) combining 8 active ingredients (such as “metformin,” “SGLT2i,” “GLP-1 RA,” or “fast-acting insulin”).

The feed-forward neural network with a pyramidal architecture consisted of 3 fully connected hidden layers (48, 32, and 17 hidden units) and a single-unit output layer. The leaky rectified linear unit activation function was used across the hidden layers. To prevent overfitting, a dropout rate of 5% was applied following the second and third dense layers. Optimization was performed using the Adam optimizer with a learning rate of 1 × 10^–4^, exponential decay rates of 0.9 and 0.999, and a numerical stability epsilon of 1 × 10^–7^. The model was trained over 250 epochs with a batch size of 128 using a learning rate of 1 × 10^–4^. To ensure training stability and mitigate exploding gradients, gradient norm clipping was applied. The final architecture included a total of 5219 trainable parameters. The neural network was trained using synthetic data to reproduce the rules with more than 85% accuracy. To incorporate additional factors not covered by the guidelines (eg, 4 CGM-derived glucodynamics), white noise features were added to the neural network input.

Hyperparameters were selected in 2 stages. First, based on synthetic data, a grid search identified the minimal model configuration that achieved at least 80% per-patient accuracy in reproducing guideline recommendations on a validation set without overfitting. Given this configuration, optimization hyperparameters were chosen based on the training and validation per-patient accuracies. Second, these values were used as the center of a grid search for transfer learning optimization hyperparameters. This selection was performed by tracking the trade-off evaluated on the training set between reproducing guideline recommendations and adapting to real-world outcomes.

The clinical data were extracted from 6715 anonymized outpatient records spanning 2009 to 2023 that met the following inclusion criteria: a diagnosis of type 2 diabetes (3529 individuals excluded), absence of GAD-65 antibodies (65 individuals excluded), no diagnosis of type 1 or type 3 diabetes (105 individuals excluded), and recorded weight and HbA_1c_ measurements at both the start and end of a treatment regimen (as both measurements are required at the specific time window; 2483 individuals excluded). Most treatment episodes were concentrated in the later years of the dataset, with the highest proportion observed in 2021 and 2022. The resulting dataset included 853 treatment regimens from 533 patients, with an average age of 60 (SD 12) years, BMI of 33 (SD 7) kg/m^2^, and HbA_1c_ of 7.8% (SD 1.7%), where the values refer to measurements taken on average within the 3 months preceding the treatment start date. In that cohort, 61% (522/853) also had hypercholesterolemia, 46% (392/853) had chronic kidney disease (CKD), and 14% (124/853) had heart failure. The therapies were the following: 41% (353/853) first-line noninsulin glucose-lowering treatment, 37% (314/853) insulin treated, 17% (144/853) monotherapy or dual therapy, and 5% (42/853) triple noninsulin glucose-lowering agents. Drug dosages and pharmacological subclasses were not considered in this analysis. Only treatment regimens lasting more than 90 days were included.

To evaluate selection bias associated with CGM use, the baseline covariates between CGM and non-CGM samples were compared using standardized mean differences (SMDs), with an SMD of 0.25 or higher indicating imbalance. Glycemic control (HbA_1c_ level; SMD 0.08) and BMI (SMD 0.12) were well balanced between groups, indicating that CGM and non-CGM samples did not differ meaningfully in overall metabolic phenotypes. eGFR (SMD 0.32) and age (SMD 0.31) showed moderate imbalance, with CGM users being slightly older (mean age 63, SD 12 years vs mean age 59, SD 12 years) and having lower eGFR (mean 72, SD 22 mL/min/1.73 m^2^ vs mean 79, SD 19 mL/min/1.73 m^2^) than non–CGM users. The difference in age and a shift from normal to mildly reduced eGFR (CKD stage 2 in both groups) does not correspond to a distinct treatment indication in diabetes management.

A subset of the records also included CGM data and was further enriched with 4 state-specific glucodynamics coefficients (defined as the rate of glucose uptake by insulin-dependent tissues [K_g_], net balance between hepatic glucose output and insulin-independent glucose uptake by the brain [T_g_], apparent first-order clearance rate for insulin [K_i_], and endogenous insulin production rate in the presence of glucose [V_i_]) obtained from a Gauss-Newton fit of Lotka-Volterra equations describing the evolution of glucose (dG/dt = –[K_g_ × G × I] + T_g_ + R_α_[t, w]) and insulin (dI/dt = –[K_i_ × I] + V_i_ × G + R_i0_) subject to the observed peaks corresponding to food intake (R_α_[t, w] = Σ_k_ u_k_ M_α_[(t-t_k_)/w, w]) [20]. Patients were included in the analysis if they had a minimum of 1 week of CGM tracking, with at least 70% of measurements during that period.

The raw glucose time series were processed using the following computational pipeline. Initial noise reduction was performed to smooth signal noise. The meal information was automatically detected from the glucose values and incorporated into the system of differential equations. The results of the numerical integration were fitted to the time series. An optimization was carried out to minimize the root mean squared error. The best-fitting set of glucodynamics coefficients for each patient was selected for the neural network input. Samples with an error exceeding a threshold were excluded from the analysis.

Continuous features were minimum-maximum normalized to scale all neural network inputs to the interval (0, 1). To prevent data leakage and increase portability, the boundaries for the minimum-maximum normalization used predefined ranges from domain knowledge. Categorical variables were one-hot encoded. Missing data were observed only in CGM samples (123/853, 14% of individuals had CGM measurements at 90 days prior to treatment start in the absence of therapy) and low-density lipoprotein cholesterol measurements (127/853, 15% of missing values). Missing features were set to 0, with a sentinel feature added to account for their absence.

After splitting the data into training (n=597, including 91/597, 15.2% with CGM data) and testing (n=256, including 32/256, 12.5% with CGM data) sets using a fixed random seed and labeling factors that were not available in the data, a neural network was trained (TensorFlow [21]) to predict the success of HbA_1c_ reduction, defined as an absolute reduction of 0.3% or higher (for HbA_1c_≥6.5%) without weight gain in patients with a BMI of 28 kg/m^2^ or higher. The baseline window for HbA_1c_ and weight parameters was defined as the 365 days preceding the initiation of therapy. The follow-up window was defined as the 365 days following therapy initiation. Information about adherence, rescue therapy, lifestyle interventions, and discontinuation was not accounted for in the treatment evaluation. When multiple measurements were available within a window, the measurement closest to the relevant date (treatment initiation for the baseline value and the end of the follow-up period for the follow-up value) was selected.

To verify that the random split did not introduce selection bias between the 2 subsets, feature-level alignment for all clinical variables was confirmed using 2-sample Kolmogorov-Smirnov tests. In addition, an adversarial validation procedure was conducted using a random forest classifier (100 estimators) trained via 5-fold cross-validation to differentiate between the training and testing subsets.

Splitting was performed at the treatment regimen level as the model was designed to predict the next treatment step depending on a patient’s clinical history. Each record reflected the clinical history up to the time that the regimen was initiated, with a new sample added only when a treatment type changed in the patient’s treatment plan, with a consequent metabolic phenotype update. Patient-level splitting was not used because, given the sample size, it would have introduced substantial imbalance in comorbidity and phenotype distribution (eg, BMI and CKD status) between the training and testing sets.

### Statistical Testing

During training, the accuracy measured against the guidelines dropped from 85% to 67%, whereas the dual-optimization forecasting capability gradually increased from 53% to 62%. A game theory concept known as Shapley values [22] and their kernel Shapley additive explanations approximation [23] were computed and stored for all predictions, mapping out a disentangled feature space that is used to define digital twin cohorts. Patients were mapped in Shapley value space rather than clinical feature space, allowing for comparison based on their model predictions and the clinical features driving those predictions.

Approximately one-third of the individualized recommendations in the test set (112/256, 44%) differed from guideline rules. Each was evaluated for statistical support to make sure that the forecast drew from cases that were likely to share similar outcomes rather than unsupported extrapolations (neural network hallucinations). This was carried out by defining a hypersphere centered on the prediction of interest and using the Manhattan distance to count similar cases falling within a specified radius in Shapley value space. Patient similarity was measured using the Manhattan distance, which is more appropriate to high-dimensional spaces than the Euclidean distance. The cumulative number of neighbors first increased quadratically until an inflection point was reached defining a digital twin cohort with similar characteristics. Rather than using a fixed radius, the hypersphere boundary was determined dynamically through an inflection point analysis of the relationship between radius size and cohort density, identifying the point at which further expansion began to include dissimilar patients. Simple proportion testing was finally used to compare the outcomes of digital twins under different therapies, rejecting a null hypothesis that individualized alternatives offer no benefit over guidelines with a chosen *P* value below .05 after Benjamini-Hochberg correction for multiple comparisons. To account for sparse regions of the feature space, a minimum cohort size threshold was applied, and hyperspheres containing fewer than 10 patients were excluded from subsequent proportion testing.

To evaluate confounder balance within cohorts, SMDs were computed for the baseline covariates defining metabolic phenotypes (HbA_1c_ and BMI) in each cohort using the same 0.25 threshold for imbalance as above. Therapeutic regimen decisions were guided by categorical clinical thresholds rather than continuous values. Therefore, an SMD exceeding 0.25 was considered clinically meaningful only when it corresponded to a shift across a clinically defined category boundary, defined by a BMI threshold of 28 kg/m² and HbA_1c_ thresholds of 6.5%, 8.5%, and 10%. Cohorts meeting this criterion were excluded from the analysis. Imbalances within a single clinical category (eg, both cohorts classified as severely uncontrolled; HbA_1c_>10%) were kept as such numerical differences do not reflect different treatment indications.

### Ethical Considerations

This retrospective study performed a secondary analysis of an existing anonymized dataset shared by collaborating investigators for the development and evaluation of neural network models. The dataset had been anonymized before it was shared with the research team, and the research team had no access to identifying keys. In accordance with the Swiss federal act on research involving human beings (Human Research Act; SR 810.30), research projects involving anonymized health-related data do not fall within the scope of the act and are therefore exempt from the requirement for ethics committee approval. This retrospective study reports research based on clinical records that are not openly accessible and cannot be used to identify individuals.

## Results

### Overview

Dual glycemic and weight targets were reached in 51.2% (131/256) of cases in the test set. Clinicians strictly adhered to the guidelines in 14.5% (37/256) of cases, for a slight improvement in outcomes to 54% (20/37), where the HbA_1c_ target was achieved in 73% (27/37) of cases and the BMI target was achieved in 70% (26/37) of cases. After selecting the real outcomes that coincided with the recommendations of the AI model, the model achieved the dual optimization target in 65% (22/34) of cases when including glucodynamics parameters (the HbA_1c_ target was achieved in 24/34, 70% of cases, and the BMI target was achieved in 28/34, 82% of cases) and in 58% (21/36) when those were left out.

To assess the statistical uncertainty of the observed treatment effects, we performed a permutation test (10,000 permutations) comparing outcomes between guidelines and AI model recommendations including glucodynamics parameters. The observed mean outcome gain was 9.8% (*P*=.48), and among the subgroup of more clinically complex patients (BMI≥28 kg/m^2^; HbA_1c_≥8%), it was 24.3% (*P*=.41). While neither reached statistical significance at this sample size, a consistent effect was observed across both the full cohort and the subgroup of more complex patients.

Our model followed guideline-based recommendations in 144 decisions; involving patients at an earlier disease stage or with better glycemic control than the average (82/144, 57% at therapy initiation; mean baseline HbA_1c_ 7%, SD 1.19% and BMI 34, SD 7 kg/m^2^) often resulted in monotherapy (GLP-1 RAs: 39/144, 27%; SGLT2 is: 19/144, 13%; metformin: 14/144, 10%). Fifty-five recommendations were excluded from testing due to insufficient data to form meaningful cohorts and perform individual statistical tests (mean baseline HbA_1c_ 8.92%, SD 2.35%; mean baseline BMI 31.46, SD 5.46 kg/m^2^).

When the decisions became more complex and could be enriched with CGM-derived glucodynamics parameters, the model identified 69 precision medicine recommendations that differed from the guidelines and were eligible for statistical testing against the guideline recommendations, 39 (56.5%) of which had statistically significant digital twins where the no-benefit hypothesis of the alternative over the guidelines could be rejected (*P*<.05). Further enriched with glucodynamics, deviations from the guidelines even had 1.8 times greater likelihood of simultaneously reducing HbA_1c_ level and managing weight.

To evaluate confounding by indication in the analysis of the model including glucodynamics parameters, the baseline characteristics of concordant (AI recommendation followed) and discordant (AI recommendation not followed) groups were compared using SMDs. Concordant individuals had higher mean baseline HbA_1c_ levels (8.48%, SD 1.7% vs 7.58%, SD 1.7%; SMD 0.53) and slightly lower BMI (31.9, SD 6.6 vs 33.5, SD 6.8 kg/m^2^; SMD –0.25). Age (SMD 0.1), eGFR (SMD –0.13), low-density lipoprotein cholesterol (SMD 0.16), and CKD diagnosis (SMD 0.08) were balanced between groups. This suggests that concordant patients were not clinically simpler cases as they had worse baseline glycemic control.

Because the hypersphere was determined dynamically for each patient using a localized inflection point, cohort sizes varied. Across all patient reassignments, cohort sizes for the guideline recommendation evaluation ranged from 11 to 93 patients, with a mean cohort size of 45 (SD 19) peers per hypersphere. For the precision medicine evaluation, cohort sizes ranged from 15 to 109 patients, with a mean cohort size of 38 (SD 24) peers per hypersphere.

Across sensitivity perturbations of cohort size (±10%-25%), cohort membership showed moderate stability (mean Jaccard index 0.669 unweighted and 0.679 weighted by cohort size). Weighting effect pairs by cohort overlap produced results similar to the unweighted analysis (Spearman *r*=0.737 and overlap-weighted Pearson *r*=0.784), with effect direction consistent in 97.3% of pairs (97.7% after weighting).

The largest group among the 69 therapy reassignments was from GLP-1 RAs in combination with insulin to GLP-1 RAs alone (16/28, 57.1% statistically significant recommendations). The second largest was switching from insulin to GLP-1 RAs (5/5, 100% statistically significant recommendations). The remaining reassignments were to replace GLP-1 RAs with SGLT2is in combination with insulin (5/5, 100% statistically significant recommendations) and replace SGLT2is with metformin in combination with insulin (5/5, 100% statistically significant recommendations). In contrast, when glucodynamics parameters were artificially switched off, the analysis identified 53 recommendations, 26 (49.1%) of which were statistically significant. Even though our study was limited by sample size, it shows that the absence of glucodynamics may reduce the capacity to tailor therapies, especially for the replacement of GLP-1 RAs with SGLT2is (1 case) and of SGLT2is with metformin (3 cases) in combination with insulin.

The primary advantage of glucodynamics parameters (K_x_, T_g_, K_i_, and V_m_) over sample statistics such as the glucose management indicator and time in range (percentage of time that glucose values are within the target range of 3.9-10.0 mmol/L) is the timely knowledge of a balance that has long-term consequences when decisions are made close to the boundaries. For example, a patient with 74% time in range and 6.8% HbA_1c_ will have guidelines recommending metformin even as the glucodynamics parameters indicate high glucose variability that justifies a more intensive treatment with SGLT2is to stabilize HbA_1c_ levels and weight.

A closer look at the Shapley values (not shown in a figure) suggests that, when the insulin clearance rate K_i_ is not high and there is no SGLT2i intolerance, the digital twin signature of individualized reassignments tends to better match patients for whom the guidelines recommend metformin, SGLT2is, and insulin; this, however, without any simple triplet combination of 23 factors standing out so that it is not possible to distill a simple model.

A more detailed knowledge of glucodynamics can noticeably help de-escalate or fine-tune therapies. For example, Figure 1 shows a patient for whom the guidelines recommended adding GLP-1 RAs with long-acting insulin at the age of 33,250 days because of a previous rapid-acting insulin therapy, a BMI of 28 kg/m^2^ or higher, and a glucose monitoring indicator exceeding 7.0%.

**Figure 1.**
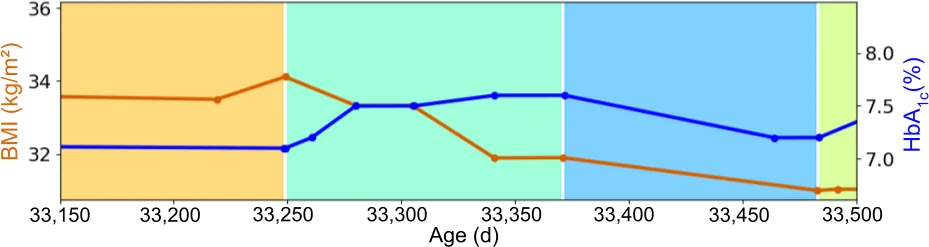
Medical history of an individual showing the evolution of hemoglobin A_1c_ (HbA_1c_) levels (in blue, right) and BMI (in orange, left) in the presence of different therapies (colored background), including metformin + insulin (yellow), metformin + GLP-1 RA + insulin (green), metformin + GLP-1 RA (blue), and metformin + SGLT2i (green)..

This combination led to a rapid HbA_1c_ increase from 7.2% to 7.6%, leading a skilled clinician to drop insulin entirely at around 33,370 days of age, effectively stabilizing weight and reducing HbA_1c_ back to 7.2%. Using only data available at that time, our neural network drew the same conclusions at 33,260 days of age, justifying the recommendation with sufficient natural insulin production and high insulin clearance, as illustrated by the Shapley values in Figure 2.

**Figure 2.**
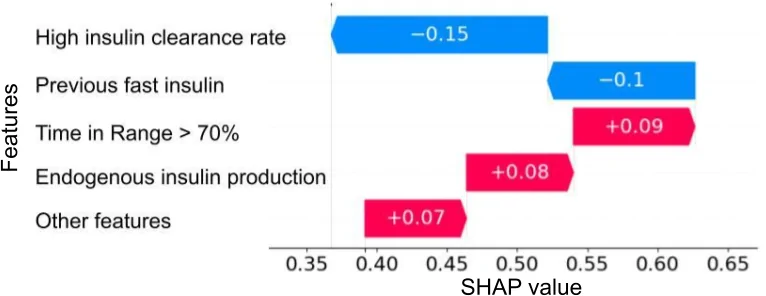
Shapley additive explanations (SHAP) values explaining how glucodynamics, such as high insulin clearance and sufficient endogenous insulin production that can be inferred from continuous glucose monitoring, lead the neural network to drop insulin from the guideline recommendations for a patient, leaving only metformin and a glucagon-like peptide-1 receptor agonist.

The physiological mechanism is not clearly understood and, in absence of a causal relationship, the possibility that the evolution observed in Figure 1 results from confounding factors that were not controlled for (eg, lifestyle changes and comorbidities, which are absent in Figure 1), either for the specific patient or for the statistically significant digital twin cohort that was used here for comparison, cannot be excluded.

To demonstrate the separation power of glucodynamics, consider a therapeutic decision for 2 patients diagnosed with type 2 diabetes with obesity (BMI≥30 kg/m^2^), dyslipidemia, and CKD who have otherwise similar controlled HbA_1c_ (5.9% and 6%) and medical histories.

Without glucodynamics input, the precision medicine model aligns with guidelines for both patients and recommends SGLT2is. Relying on CGM data available for the first patient and shown in Figure 3, it is possible to detect normal insulin sensitivity with a high endogenous insulin production rate, leading the neural network to switch to an insulin therapy. Again, owing to a skilled clinician, the switch did take place in reality, with a dramatic HbA_1c_ decrease from 14% to 7.9% and a BMI decrease from 31 to 28 kg/m^2^. In spite of current guidelines, it turns out that the second patient was also put on insulin, with a stable BMI and a slight increase in HbA_1c_ from 6.1% to 6.8% over 1 year.

**Figure 3.**
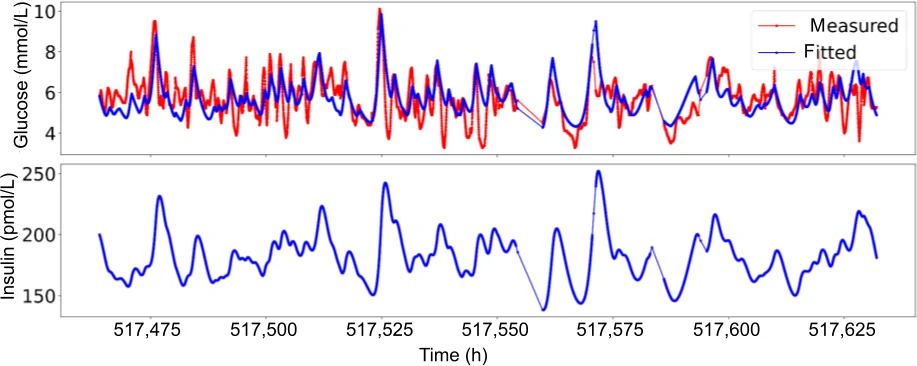
Glucose fitted with a root mean squared error of 0.84, resulting in glucodynamics parameters K_g_=0.00174, T_g_=1.30, K_i_=0.747, and V_i_=24.9 and insulin generated for the first patient, where the recommendation of the neural network was to switch from sodium-glucose cotransporter 2 inhibitors to insulin at 517,632 hours.

### Clinical Evaluation

A usability study was conducted with 12 general practitioners (GPs) and 12 endocrinology specialists to evaluate how deviations from guidelines were received for 3 groups of deidentified patient records with different clinical profiles and where the AI-generated treatment recommendations deviated from guidelines that were current at the time of the evaluation. To maintain a focus on the broader perspective, insulin dosage was pooled into 3 categories: basal, bolus, or basal-bolus. For each case, the physicians were asked to compare guideline treatment recommendations with the recommendations from the AI model. Differences in opinion, acceptance of AI-generated suggestions, and qualitative feedback were recorded. In the clinical evaluation setting, multiple comparison correction was not applied, reflecting how the system is intended to be used in practice: each patient’s statistical test is interpreted independently at the point of care rather than as part of a prespecified batch of simultaneous comparisons.

Group 1 involved 6 patients with a mean age of 59.1 (SD 9.5) years, a median BMI of 28.7 (IQR 28.7-29.6) kg/m^2^, a mean HbA_1c_ of 7.3% (SD 0.4%), and a median total daily insulin dose (TDD) of 34 (IQR 16-36) units. GPs expressed skepticism about replacing GLP-1 RAs with SGLT2is (in combination with both metformin and insulin), citing high costs and potential side effects. While some acknowledged the AI model’s recommendation as medically sound, most preferred continuing GLP-1 RA therapy and stopping insulin cautiously. Specialists generally agreed with these concerns.

Group 2 included 7 patients with a mean age of 53.7 (SD 10.6) years, a median BMI of 31.4 (IQR 28.8-36.8) kg/m^2^, a mean HbA_1c_ of 7.6% (SD 1.1%), and a median TDD of 20 (IQR 20-36) units. In this group, GPs described the AI model’s recommendation to replace insulin with SGLT2is (in combination with both metformin and GLP-1 RAs) as useful but preferred a gradual insulin tapering approach. Some questioned whether the insulin dose was too high to justify a discontinuation of insulin. Specialists agreed and suggested that bariatric surgery might be considered for some patients.

Group 3 involved 5 patients with a mean age of 66.8 (SD 7.2) years, a median BMI of 28.8 (IQR 28.7-35.6) kg/m^2^, a mean HbA_1c_ of 8.3% (SD 1.0%), and a median TDD of 20 (IQR 20-55) units for whom the guidelines recommended decreasing insulin in a therapy combining GLP-1 RAs and insulin. In this scenario, GPs were more hesitant to follow the AI model’s suggestion to withdraw medications, considering insulin discontinuation appropriate only in palliative contexts. Specialists echoed these reservations, noting that the patients’ HbA_1c_ levels were too elevated to consider stopping insulin altogether.

Two statistical analyses were performed to examine factors influencing acceptance of AI model recommendations. First, no statistically significant relationship was found between the TDD and the likelihood of accepting the AI-generated suggestions. Second, there was no significant association between the physician’s role (GP vs specialist) and their willingness to follow the AI model’s advice. These findings suggest that the decision to accept or reject AI-generated recommendations was not driven by objective metrics such as insulin dosage or by physician specialty.

In summary, the analysis demonstrated that physicians engaged thoughtfully with the AI tool and often viewed its suggestions as clinically reasonable or as the most adequate recommendation. However, final treatment decisions were guided primarily by individual clinical judgment; patient-specific context; and practical considerations such as drug cost, safety, and feasibility. Although the AI system’s recommendations were not routinely followed, its potential to support human decision-making in diabetes management was recognized. The absence of statistically significant predictors for AI acceptance further underscores the nuanced and individualized nature of medical decision-making in clinical practice.

## Discussion

The complexity of therapeutic decision-making in type 2 diabetes can be overwhelming, even for specialists, and a minority of the decisions in this study were made in full adherence to clinical guidelines, whereas only 51.2% (131/256) of the therapies achieved dual glycemic and weight optimization targets. Learning from real-world evidence outcomes in the form of digital twins, by individualizing therapies, this fraction can be improved to 58% (21/36) with commonly available features and even to 65% (22/34) when adding glucodynamics parameters from CGM recordings.

Trained on guidelines, the algorithm is portable and uses data in a parsimonious fashion to create digital twin cohorts with alternative therapies for which a no-benefit hypothesis can be rejected with a suitably low *P* value. Personalized treatment proposals consider individual factors including CGM-derived glucodynamics parameters as well as risks and comorbidities, with nuances that may be difficult for a human to incorporate.

A limitation of our work is the lack of personalization of drug dosing and distinctions between subclasses of medications (eg, different types or dosages of GLP-1 RAs), which could not be addressed with the limited amount of data available in this study. In addition, important clinical considerations such as cardiorenal comorbidities were not addressed in this study despite their established relevance to metabolic treatment in type 2 diabetes. To address these limitations, future work could follow an approach that includes using a larger and more detailed dataset; enhancing the model to incorporate additional medication-related factors; testing the model on diverse patient groups to confirm its accuracy; and, finally, assessing its real-world effectiveness through a clinical trial. To tackle frequently associated comorbidities and complications, future research could also incorporate cardiovascular and renal outcomes into the optimization.

Another limitation of this study is its retrospective design; treatment assignment was not randomized, and the analysis is vulnerable to selection bias. Therefore, prospective external randomized studies are required to fully address these biases and confirm the clinical benefit of the precision medicine framework. The first step might be a silent evaluation phase during which the model processes live clinical data and generates recommendations without influencing patient care to assess model accuracy and patient safety. A monitoring workflow could also be established to assess model performance across demographic subgroups, including age, biological sex, body weight, and ethnicity.

After testing for safe integration into clinical practice, the model is intended as a clinical decision support tool that can be integrated directly into electronic health record workflows, providing clinicians with a data-driven second opinion during patient consultations. To improve the transparency of the recommendations, predictions can be displayed with an explainable interface of Shapley values that highlights the patient-specific factors that contributed most to each prediction. The clinical guideline recommendations would be displayed along with this, helping clinicians understand how the recommendation relates to established standards of care. This provides a summary of the patient’s medical profile and an interpretation of the recommendation. However, final treatment decisions will always remain with the treating clinician, who can evaluate the model’s recommendations in the context of their clinical judgment and the patient’s preferences.

In conclusion, we propose a model that integrates clinical guidelines with routine clinical data and CGM-derived glucodynamics to identify optimal glucose-lowering therapies for HbA_1c_ and body weight goals for people with type 2 diabetes. These findings do not establish statistically significant clinical benefits but support the feasibility of this approach.

## Abbreviations

CGM: continuous glucose monitoring
CKD: chronic kidney disease
eGFR: estimated glomerular filtration rate
GLP-1 RA: glucagon-like peptide-1 receptor agonist
GP: general practitioner
HbA_1c_: hemoglobin A_1c_
SGLT2i: sodium-glucose cotransporter 2 inhibitor
SMD: standardized mean difference
TDD: total daily insulin dose
